# Healthy Aging Biomarkers: The INSPIRE’s Contribution

**DOI:** 10.14283/jfa.2021.15

**Published:** 2021-04-24

**Authors:** I. Ader, L. Pénicaud, S. Andrieu, J. R. Beard, N. Davezac, C. Dray, N. Fazilleau, P. Gourdy, S. Guyonnet, R. Liblau, A. Parini, P. Payoux, C. Rampon, I. Raymond-Letron, Y. Rolland, P. de Souto Barreto, P. Valet, N. Vergnolle, F. Sierra, B. Vellas, Louis Casteilla

**Affiliations:** 1 RESTORE, UMR 1301-Inserm 5070 Etablissement Français du Sang-Occitanie (EFS), Inserm 1031, University of Toulouse III, National Veterinary School of Toulouse (ENVT), CNRS, Toulouse, France; 2 Inserm UMR 1027, Toulouse, France; 3 University of Toulouse III, Toulouse, France; 4 Department of Epidemiology and Public Health, CHU Toulouse, Toulouse, France; 5 Centre of Excellence in Population Ageing Research, University of New South Wales, Sydney, Australia; 6 Centre de Recherches sur la Cognition Animale (CRCA), Centre de Biologie Intégrative (CBI), CNRS, University of Toulouse III, Toulouse, France; 7 Institut des Maladies Métaboliques et Cardiovasculaires, Inserm/Université Paul Sabatier UMR 1048 - I2MC 1 avenue Jean Poulhès BP, 84225 31432 Toulouse Cedex 4, France; 8 Infinity - Institut Toulousain des Maladies Infectieuses et Inflammatoires, CNRS U5051, INSERM U1291, University of Toulouse III, Toulouse, France; 9 Gérontopôle, Department of Geriatrics, CHU Toulouse, Toulouse, France; 10 ToNIC, Toulouse NeuroImaging Center, Université de Toulouse III, Inserm, UPS, Toulouse, France; 11 IRSD, Université de Toulouse, INSERM, INRA, ENVT, Université de Toulouse III, U1220, CHU Purpan, CS60039, 31024 Toulouse, France; 12 Institut du Vieillissement, Université de Toulouse, CHU Toulouse, Toulouse, France

**Keywords:** Biomarkers, healthy aging, frailty, dependence

## Abstract

The find solutions for optimizing healthy aging and increase health span is one of the main challenges for our society. A novel healthcare model based on integration and a shift on research and care towards the maintenance of optimal functional levels are now seen as priorities by the WHO. To address this issue, an integrative global strategy mixing longitudinal and experimental cohorts with an innovative transverse understanding of physiological functioning is missing. While the current approach to the biology of aging is mainly focused on parenchymal cells, we propose that age-related loss of function is largely determined by three elements which constitute the general ground supporting the different specific parenchyma: i.e. the stroma, the immune system and metabolism. Such strategy that is implemented in INSPIRE projects can strongly help to find a composite biomarker capable of predicting changes in capacity across the life course with thresholds signalling frailty and care dependence.

## Glossary

### Aging

The result of the balance between biological damage accumulation and compensatory mechanisms (1). As time passes, the compensatory mechanisms become less and less effective, which leads to more and more damage, a gradual decrease in physiological reserves and, phenotypically, this manifests as aging.

### Healthy aging

Healthy aging is defined by The World Health Organization (WHO) as the process of developing and maintaining the functional ability that enables well-being in older age, including not just absence of disease, but also happiness, satisfaction and fulfillment (2). This requires to consider health span as final outcome instead of life span that corresponds to the duration of life whatever the quality of this life.

### Robust

When an individual displays a maximal intrinsic capacity and resilience, she/he is considered as robust.

### Frailty

Frailty is a progressive age-related clinical condition characterized by a deterioration of physiological capacity leading to an increased vulnerability of the individual (3) and a higher risk of having poor health as well as a faster entry into care dependence (4). Frailty status occurs when the mobilization of the physiological reserve is not able to overcome the challenge (see adaptive capacity and resilience).

### Intrinsic capacity (IC)

IC is the composite of 5 major physiological and mental capacities of an individual that can be assessed in a day-to-day environment (5, 6). Taking into account data from large cohort studies (7, 8), the WHO has suggested 5 key domains of capacity to maintain autonomy: sensorial (vision, audition), locomotor, cognitive, psychological and underlying physiological/cellular processes (9). These domains influence each other and are in turn influenced by environmental determinants.

### Functional ability

Comprises those health-related attributes that enable people to be able to do what they want to do. This ability comprises not just the intrinsic capacity of the individual, but also the interactions between the individual and their environment.

Adaptive capacity, reserve and resilience: For a function, the ability to handle stress depends on the ability to mobilize adaptive capacity. For an individual, reserves are her/his maximal adaptive capacity. When adaptive capacity is more related to a physiological perspective, the term resilience is more generic and is coming from psychological field. At any time when the reserve exceeds the required adaptation capacity necessary to face a challenge, the individual is resilient. Frailty takes place as soon as these reserves become limited and resilience is diminished, resulting in a high sensitivity to any challenge that could lead to dependency (10).

### Geroscience

Geroscience aims to identify the underlying molecular causes of chronic diseases and aging-related conditions. It focuses on hallmarks of the aging process, irrespective of the tissue, since most of these hallmarks are molecular and not specific to a given cell type. For example, a large literature is emerging that indicates the importance of cellular senescence in defining the aging phenotype (11).

### Senescence

Irreversible form of long-term cell-cycle arrest, caused by excessive intracellular or extracellular stress or damage. Senescent cells secrete a panel of molecules deleterious for surrounding cells.

## The challenges of healthy aging and the search for biomarkers

While life expectancy has increased in recent decades, healthy life expectancy has not increased to the same extent, which means that people are living more years with functional losses generating crucial socioeconomical issues (12). As proposed by the WHO, a novel healthcare model must be privileged focused on heathy aging (glossary). This new paradigm requires an integrative and multi-disciplinary view based on the functional ability of individual to be able to do what they have reason to value. This corresponds to an interplay between the intrinsic capacity (IC, glossary) of each individual with its own environment. Intrinsic capacity can be described as the interactions between 5 domains (cognition, psychosocial, locomotion, sensory, vitality) that permits each individual to be autonomous. Consistent with this view, recent analysis suggests that quantifying an individual IC is a more useful predictor of future care dependence that the number of clinical morbidities an individual may be experiencing (9). Altogether this requires a shift of current research and healthcare models generally reactive disease-and organ centered towards the maintenance of optimal functional levels defining preventive and function-centered view. In this perspective, identifying frail persons (glossary) and especially when their IC declines and their resilience is limited (pre-frail state, glossary), as well as monitoring their individual evolution in order to propose solutions to maintain or recover IC and to prevent dependency is crucial. Pre-frailty was identified as a potentially reversible condition. Having this function-centered approach in mind, aging can be understood as the result of a subtle and progressive dysregulation of different balances that change overtime even at infra-clinic level rather than the result of a sudden appearance of one molecular or cellular defect. There is great individual diversity in the rate of this deterioration. This makes healthy aging research particularly tricky as well as in need of new multidisciplinary and global approaches to cover this unmet clinical need (13).

While chronological age (civil age, date of birth) is an easy-to-determine number and only reflects the time a person has spent on Earth, no precise definition is yet available for biological age. The basic idea behind biological aging is that aging occurs as damage to various cells and thus tissues in the body, which accumulate gradually with time and decrease the capacity to handle stress. It is the result of the interactions between intrinsic (eg. genetic, epigenetic, physiological…) and extrinsic (eg. lifestyle, infections…) factors that vary individually. In consequence, biological age differs from individual to individual for the same chronological age and thus the kinetics and slope of the biological aging process are unique to each of us (14). Very recently, the Snyder’s team has started to decipher certain elements that could explain biological age (15). They defined different types of biological aging patterns in different individuals, termed “ageotypes”. They discovered that people tend to fall into one of four biomolecular pathways associated with kidney, liver, metabolic and immune dysregulations. Some people fall squarely into one category, but others may meet the criteria of the four, depending on how their biological systems resists against aging. Unfortunately this work lacks long-term follow-up to assess whether these molecular phenotypes were ultimately associated with physiological failures.

Altogether, these reports imply that prediction, prevention and care must be personalized and adjusted to the individual’s biological, not just chronological age. This would be in line with the vision of 4P medicine (personalized, preventive, predictive and participatory) as proposed by Hood and colleagues (16–18). Even though biomarkers related to aging are at present widely used in medicine and a first list of 258 putative candidate biomarkers of aging was proposed by the TAME workgroup (19), identifying relevant biomarkers parameters in the context of biological aging and more specifically to the prefrail and frail state represents a new challenge, simply because those biomarkers do not fit within the disease-centred approaches of the current medicine.

## Biomarkers for healthy aging: Contribution of the Occitanie Toulouse INSPIRE initiative

**Figure 1:**
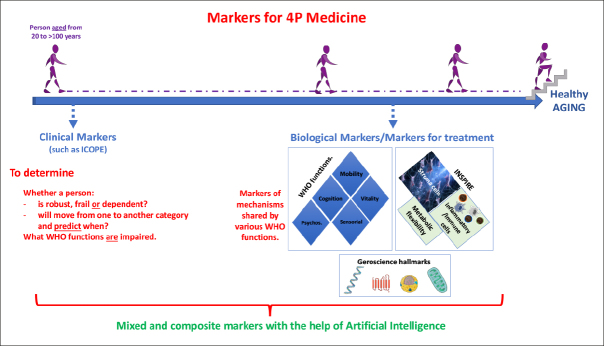
Markers for 4 P Healthy aging Medicine

It is now widely recognized that the decline of health and the appearance of aging phenotypes would be the result of simultaneous deregulations of multiple physiological systems and their interactions (20). Thus, it is likely that only the use of multiple or composite biomarkers is relevant to assess these global body disturbances. In the field of healthy aging, ideal biomarkers would be to monitor the evolution towards frailty in order to intervene as soon as possible. It would be a single composite measurement used throughout the lifetime of individuals that encompasses and combines many physiological parameters. Such biomarkers should be capable of predicting changes in capacity across the life course with thresholds signalling the evolution from one state to another. Through deconvolution into their individual components, it should suggest the most appropriate pathway of care (prevention or/and treatment) for a given individual. Furthermore, the use of these biomarkers must be able to not just assess overt expressions of capacity but the resilience of an individual’s capacities in the face of additional stressors particularly for prefrail biomarkers. The assessment of resilience is an important and challenging issue because this requires identifying the best challenge suitable to reveal the maximal adaptive capacity of the organism as a whole. Furthermore, in the best-case scenario, ideal biomarkers should be able to inform the clinician about the best-adapted care pathway for the individual (Figure 1). As usual, ideal biomarkers should also be robust, specific, as non-invasive as possible and cost-effective. Obviously, such biomarkers are much closer to fantasy than to reality, but such a definition can help the community to direct progress towards this tremendous challenge (21).

Figure 1: Clinical markers (such as those derived from ICOPE) should be assessed at first to determine the category (robust, pre-frail, frail and dependent) of individuals of various chronological ages and what WHO function(s) is (are) altered. This should help to facilitate the search for relevant biological markers taking into account both WHO functions, hallmarks of aging as well as markers based on the SIM-INSPIRE strategy. By using artificial intelligence, we believe that relevant mixed-markers of biological ages will be made available.

A large list of potential biomarkers is already available and discussed in several reviews, but these biomarkers are most often unique, studied and validated as such for specific pathologies or derived from preclinical studies that have taken changes in disease specific physiology or lifespan, rather than health expectancy. A great step forward to identify transverse elements has been taken with the development of geroscience that considers biological aging as the main risk factors of chronic diseases (22). To go beyond these concepts and in the framework of WHO perspective, we propose to implement this analysis with a physiological transverse analysis. While the current approach to biology of aging is mainly focused on parenchymal cells that are specific to each organ consistent with the current organ/disease centered view, we propose that age-related loss of function is largely determined by dysfunctions of key generic components of tissue repair processes associated with tissue renewal and particularly the defective support of parenchyma cell environment (23, 24). As such, we propose to focus much of the investigations on the stroma, the immune system and metabolism (SIM), three elements considered as the general ground that constitutes the soil supporting the emergence and the functioning of the different specific parenchyma. Inflammation and immunity both represent a warning signal and the housekeeping guardians of tissue integrity. Mesenchymal stem/stroma cells (MSC) are supportive cells of the stroma that create and maintain the functional macro-architecture of tissues, in close connexion with vascular cells. Finally, metabolism is a central component that controls any cell decision and fate. Under challenges and chronic injuries, it is well known that these three inter-related components drive cell turnover and repair outcomes. However, malfunctioning on any of these components leads to i) chronic inflammation and a low-grade immune attrition (25), generation of fibrotic cells with extracellular matrix accumulation and oxidative damage within tissue (26) and ii) accumulation of ectopic adipocytes associated with loss of metabolic flexibility and organ dysfunctions (27). These features are classically described as key elements of the aging process and age-related organ dysfunction. The INSPIRE project proposes to set-up an interdisciplinary approach that gathers clinical and scientific community working on shared bio-resources (28, 29), a fine characterization of these three inter-related components and two types of key parenchyma cells: neurons and muscle cells, corresponding to the WHO-defined functions of locomotion and cognition.

## A large panel of bio-resources facilitating the discovery of biomarkers

### INSPIRE shared bio-resources

**Figure 2:**
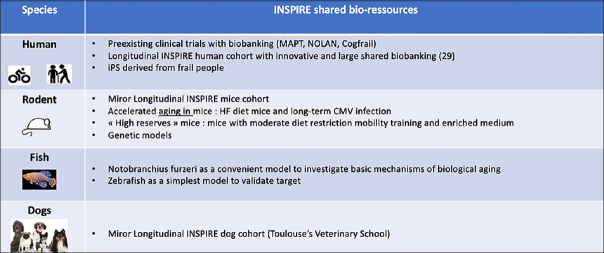
INSPIRE shared bio-resources

Identifying relevant biomarkers to detect and/or predict human frailty is challenging according to the complexity of the thematic and the human lifespan. These limits can be circumvented by the creation of a panel of biological models and samples that can cover the entire chain from the bench to the bedside and vice-versa. As starting point and at population level, the first personalized assessment to detect frailty to anticipate care dependency can be based on a non-invasive self-evaluation that can be easily deployed to a broad population in its specific environment through a digital application (30). When a problem is flagged, a team of health workers is alerted, and the individual taken care of in a more specific and detailed way guided by further assessment (Figure 1). Beside such e-cohort serving as a pool of participants to further investigations, different cohort can be implemented, including humans, dogs, mice and fish (Fig 2). In longitudinal human cohort, the longitudinal follow-up of the same individuals with a short delay between recurrent evaluations will ensure a reliability of the data and a fine description of any change in IC associated with any relevant life events (28, 30). A computational approach will permit to define predictive and prognostic biomarkers. As many experiments are not easily done in human being due both to experimental procedures, ethics considerations as well as the quite long life-time, complementary animal cohorts should help the quest of putative biomarkers.

INSPIRE aims to build and manage various cohorts in different species including human, rodents, fishes and dogs.

As the simplest but relevant model for studying biological aging, Nothobranchius Furzeri (African or Turquoise killifish) the vertebrate with the shortest known lifespan can be used (31). This animal develops very rapid aging of all its organs (4 to 6 months), with the recapitulation of all the aging hallmarks found in older-adults patients, both at the molecular and functional level (cognitive alterations, reduction of locomotion, sarcopenia onset…) (32, 33) and its health (meaning behavior) can be finely evaluated. It represents a unique animal model to quickly test and validate new hypotheses within the framework of the INSPIRE project. A murine cohort as small mammals and gold-standard physiopathological models has been designed as a mirror to the human cohort and fully described in (34). Finally, a dog cohort from adulthood to elderly represents an unvaluable tool because dog is a mammal displaying rapid aging compared to humans but with a very similar physiology including aging features and pathologies, as well as an evolution towards biological aging with IC reduction. Furthermore, it is possible to evaluate the biological and physical parameters of dogs in a manner almost identical to that of humans (physical and clinical examinations, blood and urine analyzes, medical imaging, functional tests, etc.). In addition, dogs share with their owners their environment (air, water, food, exposure to potential contaminants, …) and their lifestyle (active or sedentary) (35, 36).

In all of these animal cohorts, individuals has to be widely phenotyped and generate a large biobanking in order not only to identify putative biomarkers that can be tested and validated in human cohort but also to better investigate those mechanisms identified in the human cohorts in terms of changes in metabolism, immune and regenerative capacities.

### The challenges to identify resilience markers

Prefrail state can be determined only if the individual is submitted to a defined challenge or stress which will led to a return, or not, to a “normal” state if resilience is present, or not. Thus, to evaluate functional/physiological resilience as part of a battery of potential biomarkers of aging, we propose to carefully evaluate responses to stressors, either “lifelong challenges” or well-controlled acute stressors. In fact, different physical and psychological life events and injuries can be considered as real-life challenges, including for example both acute or chronic infections (HIV, flu, COVID-19…) but also other life events such as hip fracture or surviving the death of a spouse or a relative, during a divorce or the loss of the job. Since the follow-up of people enrolled in the INSPIRE human cohort is frequent, as soon as such challenge occurs, the stress effect on functions and biological parameters will be informed during the following evaluation. Thus, INSPIRE design can reveal physiological adaptations specific to biological aging in order to unmask biomarkers following a situation of stress.

For controlled stressors, we propose to challenge immune, inflammatory and metabolic homeostasis by an exercise corresponding to the evaluation of muscle tone (isometric measurements) coupled with intense physical exercise. Blood sampling before and just after this VO2 max challenge will allow to a better appraising of physiological reserve, as well as to test the acute response of metabolic, immune and inflammatory blood biomarkers after a maximal effort. To in vivo assess the immune system, we will monitor the magnitude and quality of the antigen-specific immune response as well as the bystander inflammatory response following administration of a vaccine included in standard care (flu, pneumococcal or zoster vaccination). Serum and peripheral blood mononuclear cells will be prospectively collected prior to, and at different time points following the vaccine shots. We will use several ‘omics’ technologies to monitor population and individual-level changes such as cell-subset phenotyping, cytokine response assays, and gene expression profiles, which will allow the longitudinal tracking of multiple immune features. These investigations can be supplemented by ex vivo challenges of precise immune cell types using stimuli targeting either innate immune cells or lymphocyte subsets. With these investigations, we aim at assessing not only the immune reserves but also immune cell global dysfunction, which may contribute by itself to premature aging, as elegantly documented in mice (37). To assess the role of supportive mesenchymal stroma/stem cells (MSC) in repair processes, the definition of a relevant physiological challenge and the way to investigate it is very tricky. The evaluation of the kinetics and quality of wound healing processes after a standardized small incision required for tissue sampling could represent a relevant test. In addition, the ability of MSC isolated from biopsies to participate in repair processes can be also indirectly evaluated through their fine characterization and their repair potential. The quality of the supportive mesenchymal compartment will be determined after an in vitro challenge induced by various inducers mimicking native molecules released after tissue injury. This corresponds to the determination of MSC multipotency including the sensitivity of these cells to differentiate towards myofibroblast as well as their pleiotropic induced paracrine activities. This will be achieved via robotized phenotyping and standardized assays using Advanced Medicinal Therapeutical Product quality control facility (www.ecellfrance.fr).

### Implementation and perspectives

An important aspect is the rapid sharing of bio-resources and data to a large multi-disciplinary community. Pending sufficient data from the living longitudinal human cohort, cross-sectional studies will be carried out to develop first lists of putative biomarker profiles with the corresponding relevant thresholds for their uses in clinical practice. Since the healthy aging field is inherently very complex, the irruption of artificial intelligence in precision medicine should be of a great help to build expert systems suitable to deploy 4P Medicine approaches (38, 39). This should largely change the field of biomarkers, moving from a single or a limited profile of biomarkers with associated relevant thresholds towards a profile of multi-modal parameters including multi-Data driven algorithms.

**Figure 3:**
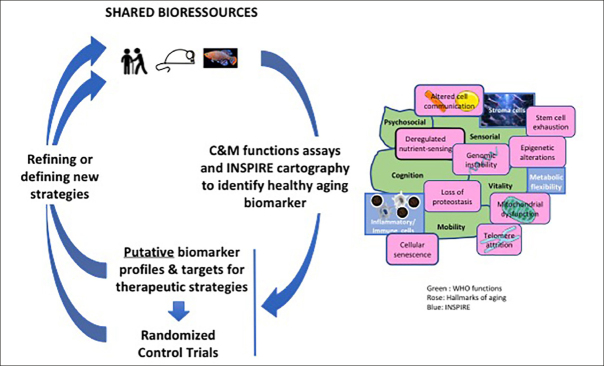
INSPIRE strategy

A complete biomarker kit to address the issue of healthy aging should contain biomarkers of pre-frailty and frailty capable of predicting individual health trajectory and the risk of care dependency. Initial biomarker sets will be tested as composite biomarkers on the INSPIRE longitudinal cohort but also will reveal putative targets for innovative therapeutic strategies that could be investigated and tested in animal models. From this first round of development and evaluation, sequential rounds will be conducted to refine and adapt these initial sets with definite validation or invalidation, inclusion of new biomarkers and a better definition of the relevant thresholds (Figure 3). Thus, the first Icope version is particularly appropriate for a large screen of individuals in primary care, but it is primarily adapted to individuals of more than 60 years old. So, if the goal is to be able to predict the risk for subsequent abnormal declines in capacity or frailty at an early age, the present evaluation should be adapted for young people prior to overt losses of capacity, it is imperative that such future measurements include parameters of resilience to challenges and stresses. For the future we can reasonably envision other types of approaches, such as artificial intelligence based image analysis methodologies. Indeed, facial recognition and locomotion gait analyses represent promising bio-visual biomarker tools, because of their accuracy and ease of implementation at the whole population level, using different digital applications.

INSPIRE will used the shared bio-resources (see figure 2) and mainly focalize on two WHO functions namely cognition (C) and mobility (M).

## Conclusion

The innovative paradigm of mixing the clinical perspective provided by the WHO innovative vision of tissue homeostasis with the geroscience perspective and modern digital tools would lead to provide a composite score to determine the biological age of each individual. Artificial intelligence will allow to achieve this objective and play a transformative role in diagnosis and therapeutic strategy. Indeed, data analysis in complex datasets comprising multiple clinical and biological variables repeated over time is extremely difficult for humans. The development of artificial intelligence in the medical field, through machine learning methodologies adapted to the need to take into account temporal data, makes it possible to envisage tools that may in the near future help the clinician to identify relevant biomarkers and to make outcome predictions. One of the keys to success in achieving this objective consists in the development of data sharing with standardized high quality data sets. This is made possible by a major scientific and financial investment by both research institutions and local authorities in INSPIRE project. It is the beginning of a long effort absolutely required to face one of the major societal challenges of our time.
